# Association of Tinnitus with Benign Paroxysmal Positional Vertigo

**DOI:** 10.3390/jcm14072473

**Published:** 2025-04-04

**Authors:** Hwa Sung Rim, Rugyeom Lee, In-Hwan Oh, Seung Geun Yeo, Sang Hoon Kim

**Affiliations:** 1 Department of Otorhinolaryngology, Head and Neck Surgery, Kyung Hee University Medical Center, School of Medicine, College of Medicine, Kyung Hee University, #1 Hoegi-dong, Dongdaemun-gu, Seoul 02447, Republic of Korea; marslover@naver.com (H.S.R.); yeo2park@gmail.com (S.G.Y.); 2 Department of Preventive Medicine, School of Medicine, Kyung Hee University, Seoul 02447, Republic of Korea; yllee19@naver.com (R.L.); parenchyme@gmail.com (I.-H.O.)

**Keywords:** tinnitus, benign paroxysmal positional vertigo, national health insurance

## Abstract

**Background/Objectives**: The purpose of this study is to investigate the potential association between tinnitus and benign paroxysmal positional vertigo (BPPV) using large-scale population data to assess the risk of developing one condition in patients who have the other condition. **Methods**: Using claims data from the National Health Insurance Corporation spanning 2008 to 2021, we conducted a comprehensive analysis to estimate the risk of developing BPPV in patients with tinnitus and vice versa. This study involved 580,531 patients with tinnitus, 572,937 patients with benign paroxysmal positional vertigo, and their corresponding controls. We used propensity score matching and statistical analyses, including Cox proportional hazard models to assess the association between these conditions. **Results**: The incidence of BPPV in patients with tinnitus was significantly higher (12.3 per 1000 individuals per year) than that of controls (5.1 per 1000 individuals per year), with an adjusted hazard ratio of 2.474. Additionally, the incidence of tinnitus was significantly higher in patients with BPPV (11.7 per 1000 individuals per year) than in controls (5.5 per 1000 individuals per year), with an adjusted hazard ratio of 2.048. Subgroup analysis showed the risk of developing BPPV in people with tinnitus, and vice versa, was higher in young vs. old people (<39 years) and in men vs. women (*p*<0.0001). These findings remained significant even after adjusting for sex, age, medical benefits, disability, and health habits. **Conclusions**: This study provides substantial evidence for a bidirectional association between tinnitus and benign paroxysmal positional vertigo, suggesting an interconnected pathophysiology. Further research is warranted to understand the underlying mechanisms.

## 1. Introduction

Tinnitus and benign paroxysmal positional vertigo (BPPV) are two common conditions that can have a significant impact on quality of life. In addition to the impairment of quality of life, both tinnitus and vertigo are associated with psychiatric symptoms [1,2]. Tinnitus is characterized by the perception of sound in the absence of an external source, often described as ringing, buzzing, or hissing in the ears [3]. Although tinnitus is primarily associated with inner ear dysfunction, growing evidence suggests that the central nervous system also plays a significant role in its pathophysiology. BPPV, on the other hand, is the most common vestibular disorder causing brief episodes of dizziness associated with changes in head position, often accompanied by nystagmus [4].

Despite their distinct primary clinical manifestations, a growing body of clinical evidence suggests a possible link between tinnitus and BPPV. Some researchers have provided valuable insights into the clinical observations and theoretical frameworks, suggesting an association between the two conditions [5]. Furthermore, tinnitus of vestibular origin, in which disturbances in the vestibular system contribute to auditory symptoms, has been recognized in clinical practice [6]. However, the precise nature of the relationship between the two conditions remains unclear primarily because of the lack of large-scale comprehensive studies investigating their association.

The epidemiology of tinnitus indicates its widespread prevalence, affecting about 10–15% of the adult population globally, with a higher prevalence in older adults and individuals exposed to occupational or recreational noise. The prevalence of tinnitus increases with age and is associated with various risk factors, including hearing loss, ototoxic medications, noise exposure, and vascular risk factors. Tinnitus significantly impacts the quality of life, leading to sleep disturbances, difficulty concentrating, and emotional distress [7].

BPPV is the most common vestibular disorder, affecting 8% of individuals experiencing moderate to severe dizziness or vertigo. Its lifetime prevalence has been reported at 2.4%, and it is more frequent in women than in men [8]. The pathophysiology of BPPV involves canalithiasis and cupulolithiasis, with posterior canal BPPV being the most common form due to the gravity-dependent nature of the posterior semicircular canal. The incidence of BPPV increases with age, peaking in the fifth and seventh decades of life. BPPV is characterized by short episodes of vertigo triggered by changes in the head position, reflecting its mechanical origin within the inner ear [4]. BPPV is one of the most common causes of vertigo, occurring when otoconia become dislodged and migrate into the posterior semicircular canal or other semicircular canals, leading to inappropriate endolymphatic flow and erroneous vestibular signals. The inner ear consists of the cochlea and vestibular apparatus, which are interconnected anatomically and functionally, sharing the vestibulocochlear nerve and a common blood supply, primarily from the labyrinthine artery. Emerging evidence suggests a potential link between BPPV and asymmetric hearing loss, with studies indicating that otoconial displacement may preferentially occur in the worse-hearing ear, potentially due to shared vascular or degenerative mechanisms affecting both the cochlear and vestibular structures [9].

The present study aimed to explore the potential bidirectional relationship between tinnitus and BPPV. Utilizing extensive claims data from the National Health Insurance Corporation in South Korea (which is the government agency managing the universal healthcare system and providing medical coverage for all residents) from 2008 to 2021, we aimed to estimate the risk of developing BPPV in patients with tinnitus and vice versa. This study employed a robust methodology, including propensity score matching, to ensure a rigorous comparison between affected individuals and the control group. By providing insights into the possible relationship between these two conditions, this study aimed to contribute valuable knowledge to the field of otolaryngology and improve the clinical management of patients with these disorders.

## 2. Materials and Methods

### 2.1. Study Design and Data Sources

Using claims data from the National Health Insurance Corporation spanning 2008 to 2021, we estimated the risk of BPPV in patients with tinnitus and the control group and the risk of tinnitus in patients with BPPV and the control group. The control groups were created using propensity score matching, ensuring a 1:1 match based on age, sex, medical history, and comorbidities to minimize selection bias. Patients with tinnitus and BPPV were each matched with controls who had never been diagnosed with the respective condition, using a 2-year washout period to exclude pre-existing cases. The data were obtained by sampling 25% of the cases based on age and sex, in accordance with the National Health Insurance Corporation’s DB provision guidelines. These data included demographic variables, health insurance claim variables, medical history variables, death, and disability. Disease definitions were based on the International Classification of Diseases (ICD)-10 codes (Tinnitus H931 and BPPV H811). The diagnostic criteria for BPPV include recurrent positional vertigo triggered by head movements, characteristic positional nystagmus observed during specific maneuvers such as the Dix–Hallpike or supine roll test, a duration of symptoms typically lasting less than one minute, and the exclusion of other potential causes [4]. The research participants of the NHIC claims database were identified by matching each disease group and the control group at a 1:1 ratio by age and sex.

### 2.2. Participants

The entire DB uses the National Health Insurance Corporation’s claims data from 2008 to 2021. The washout period was defined as 2 years from the year of first registration in the qualification data, and the risk of disease occurrence was analyzed for individuals who were not hospitalized or used outpatient services for the relevant disease. The observational period began in 2008 and continued until 31 December 2021, i.e., the end of the study period, or until death or disease occurrence. After excluding cases of medical care usage based on morbidity codes, deceased patients, and cases with missing values—which may have resulted from uncollected variables, data inconsistencies, or participant dropout—the final study included 580,531 and 712,014 patients in the tinnitus and control groups and 572,937 and 723,134 patients in the BPPV and control groups, respectively. The propensity score was applied to match the cases and controls at a 1:1 ratio.

## 3. Statistical Analysis

Basic demographic information was analyzed for frequency among the cases and controls. A chi-square test was performed for categorical variables, and a *t*-test was used for continuous variables. Prior to conducting the *t*-test, the normality of the data was assessed using the Shapiro–Wilk test and visual inspection through Q–Q plots.

The occurrence of each disease was defined using the disease code as a patient hospitalized more than once or using outpatient medical care more than once. The risk of disease occurrence was presented as a hazard ratio (HR) using the Cox proportional hazards model, with a 95% confidence interval. Subgroup analysis was conducted to assess disease occurrence based on age, sex, and health checkup variables. The variables included sex (male or female), age (continuous), disability (disabled or absent), and medical benefit status, which was a correction variable for the presence or absence of hypertension, diabetes, dyslipidemia, cardiovascular disease (CVD), and stroke. The health checkup variables included smoking status (non-smoker, former smoker, or smoker), drinking level (0 days/week, 1–2 days/week, 3–4 days/week, or ≥5 days/week), and exercise level (0 days/week, 1–2 days/week, 3–4 days/week, or >5 days/week). According to the WHO’s classification, BMI is categorized as underweight (<18.5), normal weight (18.5–24.9), overweight (25.0–29.9), and obesity (≥30.0), with obesity further divided into Class I (30.0–34.9), Class II (35.0–39.9), and Class III (≥40.0) [10]. All statistical analyses were performed using the SAS Enterprise Guide tool version 8.3 (SAS Institute Inc., Cary, NC, USA) provided by the National Health Insurance Corporation.

## 4. Results

During the 7.3-year observation period, 580,531 patients were diagnosed with tinnitus. The control group comprised 712,014 patients. Notably, a higher proportion of patients had tinnitus (259,492 men (44.7%) vs. 321,039 women (55.3%). The average age of the cohort was 52.7 years. After matching the cases and controls in a 1:1 ratio through propensity score matching, the number of patients with tinnitus and controls was 531,953 in each group. No significant differences in sex and age distribution, medical benefit status, disability status, hypertension, diabetes, hyperlipidemia, CVD, and stroke were observed between the groups. The lack of significant differences indicates that the groups are comparable (Table 1).

During the 7.0-year observation period, 572,937 patients with BPPV and 723,134 controls were sampled. After matching the cases and controls in a 1:1 ratio through propensity score matching, the number of patients with BPPV and controls was 523,276 in each group. No significant differences in sex and age distribution, medical benefit status, disability status, hypertension, diabetes, hyperlipidemia, CVD, and stroke were observed between the groups (Table 2).

**Figure 1:**
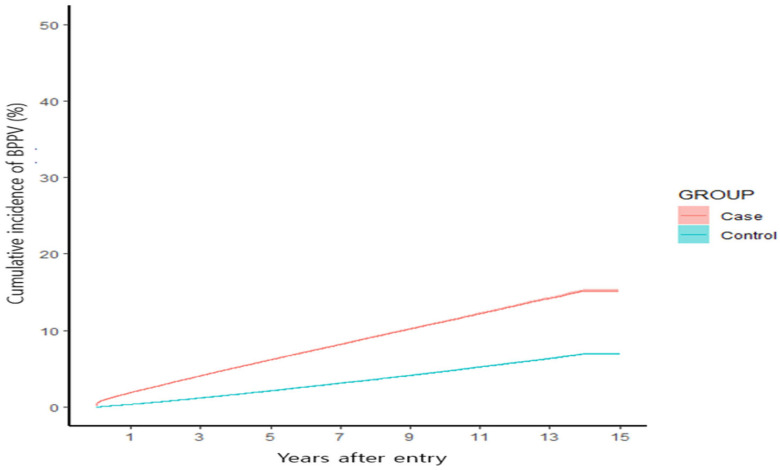
Cumulative incidence of BPPV in the tinnitus and control groups.

Based on the demographic analysis, the incidence rate of BPPV was 12.3 per 1000 individuals per year for patients diagnosed with tinnitus as the primary disease, whereas the incidence rate of BPPV was 5.1 per 1000 individuals per year for patients who had never been diagnosed with tinnitus (Figure 1).

Based on the analysis using the Cox proportional hazards model, patients with tinnitus exhibited a higher probability of developing BPPV than that of controls, with a HR of 2.474. This result was obtained after adjusting for variables, such as sex, age, medical benefits, disability, high blood pressure, diabetes, hyperlipidemia, CVD, and stroke. After applying the screening data and adjusting for smoking, drinking, exercise, and BMI, the HR was 2.249. The subgroup analysis based on age revealed that patients aged <39 years had the highest probability of BPPV occurrence (HR 3.002), followed by those aged 40–59 years (HR 2.533) and those aged ≥60 years (HR 2.269). Additionally, the probability of occurrence was higher in men (HR 2.793) than in women (HR 2.37) (Table 3).

**Figure 2:**
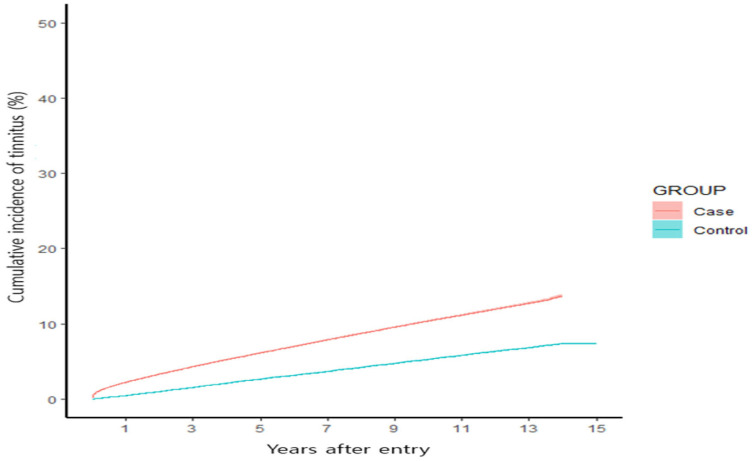
Cumulative incidence of tinnitus in the BPPV and control groups.

Conversely, according to the demographic analysis, the incidence of tinnitus was 11.7 per 1000 individuals per year for patients with BPPV as a chronic disease, whereas it was 5.5 per 1000 individuals per year for patients who had never been diagnosed with BPPV (Figure 2).

In the analysis using the Cox proportional hazards model, patients with BPPV exhibited a higher probability of tinnitus than that of controls, with a HR of 2.048. This result was obtained after adjusting for variables, such as sex, age, medical benefits, disability, high blood pressure, diabetes, hyperlipidemia, CVD, and stroke. After applying the screening data and adjusting for smoking, drinking, exercise, and BMI, the HR was 1.812. The subgroup analysis based on age revealed that patients aged <39 years had the highest probability of tinnitus occurrence (HR 2.727), followed by those aged 40–59 years (HR 2.118) and those aged ≥60 years (HR 1.814). Additionally, the probability of occurrence was higher in men (HR 2.291) than in women (HR 1.968) (Table 4).

## 5. Discussion

The findings of our comprehensive study provide a significant leap in understanding the bidirectional association between tinnitus and BPPV, suggesting a complex and intertwined pathophysiology. Utilizing a robust dataset from the National Health Insurance Corporation spanning 2008 to 2021, our analysis revealed a statistically significant increased risk of developing BPPV in individuals with tinnitus and vice versa. This bidirectional relationship underscores the potentially shared or interconnected pathophysiological basis of the two conditions, challenging the conventional perception of them as isolated disorders. While this study suggests a potential link between BPPV and tinnitus, further investigation is required to establish a definitive pathophysiological connection. Instead of focusing solely on causality, future research should explore shared risk factors and central nervous system mechanisms, particularly how vertigo-related neuroplastic changes contribute to psychiatric symptoms and potentially trigger tinnitus.

A crucial finding of our study was the notably higher incidence of BPPV among patients with tinnitus, recorded at 12.3 per 1000 individuals per year, compared to 5.1 per 1000 individuals per year in the control group. The adjusted HR for this association was 2.474. This notable difference suggests that tinnitus may serve as a risk factor or early indicator for the development of BPPV. Conversely, the incidence of tinnitus in patients diagnosed with BPPV (11.7 per 1000 individuals per year) significantly exceeded that in patients in the control group (5.5 per 1000 individuals per year), yielding an adjusted HR of 2.048. This indicates that BPPV may predispose individuals to tinnitus, highlighting the reciprocal relationship between the two conditions. Our analysis also highlighted an interesting age-related trend, with individuals younger than 39 years exhibiting the highest risk for both conditions. This finding indicates a possible heightened sensitivity or a different pathophysiological mechanism in younger individuals compared to older populations. Furthermore, this study revealed a higher incidence in men, potentially influenced by occupational noise exposure, head trauma, lifestyle factors, and genetic predispositions. Greater exposure to noise and physical strain in certain industries, along with higher smoking and alcohol consumption rates and anatomical or vascular differences, may contribute to increased susceptibility in men.

Several pathophysiological hypotheses have been proposed to explain the association between BPPV and tinnitus. The vestibulocochlear pathway dysfunction hypothesis posits that dysfunctions in the pathway, which encompasses both the cochlea (responsible for hearing) and vestibular (responsible for balance) systems, can lead to simultaneous auditory symptoms (tinnitus) and vertigo (BPPV). This is supported by the anatomical and functional proximity of these systems, suggesting that disturbances in one system can readily affect another [11,12]. Another hypothesis, otolithic dysfunction, results from dislodged otoliths in the semicircular canals, impacting cochlear sensitivity and providing a shared anatomical and functional basis for both conditions [11]. Additionally, inflammatory processes within the inner ear, potentially stemming from autoimmune disorders or viral infections, can simultaneously impact cochlear and vestibular systems, leading to the onset of both tinnitus and BPPV [13]. This suggests a role of systemic inflammation in the pathogenesis of these conditions, potentially offering targets for therapeutic interventions. Genetic predisposition is another area of interest, with certain individuals possibly being more prone to both tinnitus and BPPV than others. Identifying the specific genetic markers or mutations associated with these conditions can lead to a better understanding and potentially targeted interventions [14,15]. Shared risk factors, such as age-related degeneration [16], exposure to ototoxic medications [17], or lifestyle factors such as smoking [18] or high alcohol consumption [19], can simultaneously affect the cochlear and vestibular systems, highlighting a multifactorial etiology for these conditions.

Recent evidence suggests that in BPPV patients with asymmetric hearing loss, otoconial displacement is more likely to occur in the ear with the worse hearing threshold, highlighting the potential clinical value of an audiological assessment in evaluating BPPV laterality. This study found that threshold asymmetry was a predictive factor for BPPV in the worse-hearing ear, highlighting a potential link between auditory and vestibular dysfunctions [19]. Studies have suggested a potential link between BPPV and sudden sensorineural hearing loss as both conditions may share common pathophysiological mechanisms such as vascular insufficiency, viral inner ear damage, or labyrinthine hemorrhage. Additionally, tinnitus is often observed in patients with SSNHL and may persist even after the resolution of vestibular symptoms, further indicating a possible interplay between auditory and vestibular dysfunctions [20].

Auditory brainstem response (ABR) findings in BPPV patients suggest that auditory pathway ischemia may be involved in the pathophysiology of BPPV, and in cases accompanied by tinnitus, the localization of tinnitus may help identify the affected side [21]. The strengths of our study lie in its extensive sample size and the longitudinal nature of the data, which enhanced the reliability and validity of the findings. However, this study has some limitations. The reliance on ICD-10 codes for disease identification may not fully capture all clinical nuances. Additionally, the absence of detailed data on genetic factors, lifestyle habits, and noise exposure may have influenced the interpretation and applicability of the findings. Another limitation is the lack of comprehensive hearing status data for participants as hearing impairment may be associated with vestibular dysfunction. Future research should incorporate detailed audiometric evaluations to better understand this relationship. Furthermore, there is a possibility of BPPV being overdiagnosed as it is the most common peripheral vestibular disorder and may be considered even in cases where other causes of vertigo are present.

The bidirectional association between tinnitus and BPPV calls for a more nuanced understanding and multidisciplinary approach to its management. Our findings have substantial implications for both clinical practice and public health. Given the high prevalence of these conditions, especially in certain demographics, our study emphasizes the need for increased clinical awareness and possibly a more integrated approach to patient care.

## 6. Conclusions

Our study highlights a major bidirectional association between tinnitus and BPPV, underscoring the need for a comprehensive assessment and management of patients with these conditions. The correlation between tinnitus and BPPV is crucial not only in understanding their coexistence but also in assessing their impact on quality of life and associated psychiatric symptoms; moreover, these associations hold clinical significance not only for otolaryngologists but also for audiologists. Further research is needed to better understand the underlying pathophysiological mechanisms, which could contribute to the development of more targeted and effective treatments. Future studies, particularly those investigating the molecular and genetic bases of these disorders, as well as detailed clinical phenotyping, may help clarify the relationship between tinnitus and BPPV. Such research could provide useful insights for the scientific community and potentially improve the management of individuals affected by these conditions.

## Figures and Tables

**Table 1:** Demographics of patients with BPPV in the tinnitus and control groups.

		Total					Propensity Score Match (PSM)				
		Control Group		BPPV Group		*p*-Value (Chisq, *t*-Test)	Control Group		BPPV Group		*p*-Value (Chisq, *t*-Test)
		N	%	N	%		N	%	N	%	
Total		723,134	100.0	572,937	100.0		523,276	100.0	523,276	100.0	
Sex	Male	222,877	30.8	180,627	31.5	<0.001	162,614	31.1	162,614	31.1	>0.99
	Female	500,257	69.2	392,310	68.5		360,662	68.9	360,662	68.9	
Age	20–29	73,489	10.2	45,518	7.9	<0.001	44,424	8.5	44,424	8.5	>0.99
	30–39	115,780	16.0	79,838	13.9		77,966	14.9	77,966	14.9	
	40–49	163,970	22.7	111,383	19.4		109,236	20.9	109,236	20.9	
	50–59	159,128	22.0	135,038	23.6		125,173	23.9	125,173	23.9	
	60–69	127,314	17.6	105,418	18.4		97,838	18.7	97,838	18.7	
	70–79	70,866	9.8	72,490	12.7		58,306	11.1	58,306	11.1	
	80+	12,587	1.7	23,252	4.1		10,333	2.0	10,333	2.0	
HTN	no	385,218	53.3	303,555	53.0	0.001	279,185	53.4	279,185	53.4	>0.99
	yes	337,916	46.7	269,382	47.0		244,091	46.6	244,091	46.6	
DM	no	422,357	58.4	315,942	55.1	<0.001	259,592	56.5	295,592	56.5	>0.99
	yes	300,777	41.6	256,995	44.9		227,684	43.5	277,684	43.5	
Dyslipidemia	no	267,939	37.1	163,287	28.5	<0.001	159,831	30.5	159,831	30.5	>0.99
	yes	455,195	62.9	409,650	71.5		363,445	69.5	363,445	69.5	
CVD	no	627,472	86.8	478,240	83.5	<0.001	447,223	85.5	447,223	85.5	>0.99
	yes	95,662	13.2	94,697	16.5		76,053	14.5	76,053	14.5	
stroke	no	586,889	81.2	440,959	77.0	<0.001	412,242	78.8	412,242	78.8	>0.99
	yes	136,245	18.8	131,978	23.0		111,034	21.2	111,034	21.2	

BPPV, benign paroxysmal positional vertigo; HTN, hypertension; CVD, cardiovascular disease; DM, diabetes mellitus.

**Table 2:** Demographics of patients with tinnitus in the BPPV and control groups.

		Total					Propensity Score Match (PSM)				
		Control Group		Tinnitus Group		*p*-Value (Chisq, *t*-Test)	Control Group		Tinnitus Group		*p*-Value (Chisq, *t*-Test)
		N	%	N	%		N	%	N	%	
Total population		712,014		580,531			531,953		531,953		
Sex	Male	301,031	42.3	259,492	44.7	<0.001	234,190	44.0	234,190	44.0	>0.99
	Female	410,983	57.7	321,039	55.3		297,763	56.0	297,763	56.0	
Age	20–29	71,323	10.0	58,867	10.1	<0.001	56,923	10.7	56,923	10.7	>0.99
	30–39	102,650	14.4	72,898	12.6		71,589	13.5	71,589	13.5	
	40–49	152,898	21.5	102,651	17.7		101,385	19.1	101,385	19.1	
	50–59	162,749	22.9	134,577	23.2		126,946	23.9	126,946	23.9	
	60–69	139,735	19.6	116,175	20.0		106,930	20.1	106,930	20.1	
	70–79	71,268	10.0	75,825	13.1		59,032	11.1	59,032	11.1	
	≥80	11,391	1.6	19,538	3.4		9148	1.7	9148	1.7	
HTN	no	363,680	51.1	303,541	52.3	<0.001	282,127	53.0	282,127	53.0	>0.99
	yes	348,334	48.9	276,990	47.7		249,826	47.0	249,826	47.0	
DM	no	405,742	57.0	318,393	54.8	<0.001	300,022	56.4	300,022	56.4	>0.99
	yes	306,272	43.0	262,138	45.2		231,931	43.6	231,931	43.6	
Dyslipidemia	no	260,255	36.6	175,535	30.2	<0.001	172,162	32.4	172,162	32.4	>0.99
	yes	451,759	63.4	404,996	69.8		359,791	67.6	359,791	67.6	
CVD	no	610,801	85.8	497,337	85.7	0.06	459,769	86.4	459,769	86.4	>0.99
	yes	101,213	14.2	83,194	14.3		72,184	13.6	72,184	13.6	
stroke	no	570,990	80.2	448,659	77.3	<0.001	420,040	79.0	420,040	79.0	>0.99
	yes	141,024	19.8	131,872	22.7		111,913	21.0	111,913	21.0	

BPPV, benign paroxysmal positional vertigo; HTN, hypertension; CVD, cardiovascular disease; DM, diabetes mellitus.

**Table 3:** Hazard ratio of the incidence of BPPV in patients with tinnitus and in controls, with subgroup analysis based on age and sex.

		Onginal			PSM				
		HR	95% LCI	95% UCI	*p*-Value	HR	95% LCI	95% UCI	*p*-Value
Total	BPPV	2.477	2.444	2.51	<0.0001	2.474	2.438	2.51	<0.0001
	Control	1				1			
age < 40	BPPV	3.05	2.946	3.158	<0.0001	3.002	2.891	3.118	<0.0001
	Control	1				1			
40 ≤ age < 60	BPPV	2.609	2.557	2.662	<0.0001	2.533	2.478	2.589	<0.0001
	Control	1				1			
age ≥ 60	BPPV	2.277	2.23	2.325	<0.0001	2.269	2.218	2.322	<0.0001
	Control	1				1			
Male	BPPV	2.801	2.732	2.872	<0.0001	2.793	2.719	2.87	<0.0001
	Control	1				1			
Female	BPPV	2.353	2.316	2.39	<0.0001	2.37	2.33	2.412	<0.0001
	Control	1				1			

BPPV, benign paroxysmal positional vertigo; PSM, propensity score matching; HR, hazard ratio; CI, confidence interval; U, upper; L, lower.

**Table 4:** Hazard ratio of the incidence of tinnitus in patients with BPPV and in controls, with subgroup analysis based on age and sex.

		Original			PSM				
		HR	95% LCI	95% UCI	*p*-Value	HR	95% LCI	95% UCI	*p*-Value
Total	Tinnitus	2.037	2.01	2.065	<0.0001	2.048	2.018	2.078	<0.0001
	Control	1				1			
age < 40	Tinnitus	2.731	2.637	2.829	<0.0001	2.727	2.621	2.838	<0.0001
	Control	1				1			
40 ≤ age < 60	Tinnitus	2.157	2.114	2.2	<0.0001	2.118	2.073	2.165	<0.0001
	Control	1				1			
age ≥ 60	Tinnitus	1.801	1.764	1.84	<0.0001	1.814	1.773	1.856	<0.0001
	Control	1				1			
Male	Tinnitus	2.262	2.206	2.32	<0.0001	2.291	2.229	2.354	<0.0001
	Control	1				1			
Female	Tinnitus	1.953	1.922	1.985	<0.0001	1.968	1.935	2.003	<0.0001
	Control	1				1			

BPPV, benign paroxysmal positional vertigo; PSM, propensity score matching; HR, hazard ratio; CI, confidence interval; U, upper; L, lower.

## Data Availability

The original contributions presented in this study are included in the article. Further inquiries can be directed to the corresponding authors.
